# Continual-Learning-Enhanced CNN–Transformer Framework for Real-Time Motor-Imagery BCI in Virtual Environments

**DOI:** 10.3390/bioengineering13050536

**Published:** 2026-05-06

**Authors:** Chao-Jen Huang, Cheng-Fu Cao, Kuo Kai Shyu, Te-Min Lee, Po-Lei Lee

**Affiliations:** 1 Department of Electrical Engineering, National Central University, Taoyuan 32001, Taiwan; a5531749@gmail.com (C.-J.H.); a36210507@gmail.com (C.-F.C.); kkshyu@ee.ncu.edu.tw (K.K.S.); teminlee@gmail.com (T.-M.L.); 2 Department of Medical Research, Cathay General Hospital, Taipei 106, Taiwan

**Keywords:** motor imagery, brain–computer interface, continual learning

## Abstract

Motor imagery (MI)-based brain–computer interfaces (BCIs) provide an intuitive pathway for neural interaction and rehabilitation, yet their practical deployment remains constrained by long calibration requirements, substantial inter-subject variability, and the non-stationary nature of EEG signals. These challenges are amplified when using dry-electrode EEG, which offers superior convenience for real-world systems but produces noisier and less stable recordings than traditional wet electrodes. As a result, online or real-time four-class MI detection—especially with dry electrodes—has been explored only in a limited number of studies, underscoring an important gap in the field and the need for adaptive, intelligent models capable of coping with continuous signal drift. In this study, we propose a real-time MI-BCI framework that integrates immersive action observation (AO) in virtual reality with a continual learning strategy to manage the evolving nature of dry-EEG features. A CNN–Transformer hybrid model is first initialized through AO-enhanced pre-training and subsequently refined via online continual adaptation during user interaction. This continual learning mechanism enables the classifier to incrementally assimilate new MI patterns while preserving previously acquired knowledge, thereby mitigating the performance degradation that typically arises in extended MI-BCI sessions. Experimental results across four motor classes demonstrate improved decoding accuracy and strengthened sensorimotor activation over time, confirming the system’s capacity for user-specific and session-to-session adaptation. By addressing the rarely studied combination of dry electrodes, online four-class MI decoding, and continual learning, the proposed approach enhances MI-BCI robustness, reduces calibration burden, and supports sustainable long-term deployment in intelligent neurotechnology applications.

## 1. Introduction

Motor imagery (MI)-based classification remains one of the most challenging problems in brain–computer interfaces (BCIs), despite its central role in assistive control and neurorehabilitation. For example, Lin et al. [1] developed a VR-integrated MI training system with real-time electromyography (EMG) feedback and reported promising outcomes in post-stroke rehabilitation. Similarly, Gordleeva et al. [2] proposed an EEG–EMG hybrid human–machine interface (HMI) for lower-limb exoskeleton control, where combining leg MI with corresponding EMG signals improved both accuracy and reliability. These studies illustrate the potential of intelligent human–machine systems for motor restoration and assistive robotics. However, they also reflect a broader trend: most existing MI-BCI systems are highly engineered for specific scenarios, require extensive calibration, and often rely on controlled laboratory conditions that limit scalability in real-world applications.

In practice, MI-BCI systems still face several fundamental obstacles. Achieving stable and effective BCI control typically demands weeks or even months of user training [3], substantially increasing time and resource costs and hindering wider clinical and home deployment. Moreover, EEG signal patterns vary considerably across individuals due to physiological and anatomical differences, meaning that even for nominally identical tasks, sensorimotor activation can differ markedly between users [4]. Conventional MI paradigms—such as those used in benchmark datasets and competitions [5]—usually employ abstract symbolic cues to prompt MI. For novice users in particular, these cue-based paradigms are difficult to interpret and execute consistently without prior experience, resulting in highly variable EEG patterns across sessions and participants and limiting both classification performance and system generalizability.

To reduce training costs and enhance the consistency of neural activation, researchers have increasingly turned to Action Observation (AO) as a complementary mechanism to MI. AO refers to the engagement of the mirror neuron system when individuals observe others performing specific motor actions [6,7,8]. Prior work has demonstrated substantial overlap between the neural circuits recruited by AO and MI, even when each is executed in isolation [9]. Building on this, combined AO + MI paradigms have been shown to produce stronger cortical activation and more robust engagement of the mirror neuron system compared with AO or MI alone [10]. At the same time, virtual reality (VR) can further amplify these benefits by providing immersive, task-specific environments that reduce external distractions and promote sustained engagement [11]. For instance, Choi et al. [12] implemented a VR-based training scenario in which participants controlled a drone along a predefined path using AO + MI-driven training for an LDA classifier, followed by feedback-based control. Participants who received visual feedback of grasping hand movements significantly outperformed those without feedback, underscoring the potential of AO + MI paradigms combined with immersive feedback. More recent studies have further reported that combining VR-based action observation with kinesthetic motor imagery [13] can enhance rhythmic modulation and improve task differentiation, supporting the neurophysiological plausibility of AO-in-VR priming. Additionally, VR-enhanced AO + MI has been increasingly integrated with rehabilitation-oriented systems (e.g., exoskeleton-related frameworks), highlighting the growing interest in ecologically valid training paradigms.

Despite these advances in training design and user engagement, the core signal-processing problem remains difficult. Due to the inherently non-stationary nature of EEG and the pronounced inter- and intra-subject variability, traditional feature extraction techniques—such as Common Spatial Patterns (CSP), Wavelet Transform (WT), and Short-Time Fourier Transform (STFT) [14]—often struggle to capture discriminative, temporally stable features from complex neural data. Deep learning has therefore gained traction in the BCI community, as it can learn hierarchical, non-linear representations directly from raw or minimally preprocessed signals in an end-to-end manner. In particular, convolutional neural networks (CNNs) have demonstrated strong performance in MI classification, efficiently exploiting spatial–temporal structure in EEG and frequently outperforming handcrafted features in terms of both accuracy and generalization [15]. More recently, transformer-based architectures have further improved offline decoding performance, especially in multi-class (e.g., four-class) MI settings [16]. However, these gains are overwhelmingly reported in offline analyses under controlled conditions.

A further complication arises from the dynamic nature of the human brain. Sensorimotor rhythms (SMRs), which are widely used in MI-based BCIs, exhibit substantial variability over time and across users. Turi et al. [3] reported that SMR characteristics can be strongly influenced by experimental environment, psychological state, and underlying neural circuitry. Saha et al. [17] documented substantial intra- and inter-subject variability in EEG signals, while Morioka et al. [4] observed that even within a single individual, EEG features may vary across phases or sessions, partly due to neural plasticity and repeated MI training. This continual adaptation of the nervous system—though beneficial for rehabilitation—poses a challenge for static classification models, which are typically trained once and then deployed without mechanisms to cope with gradual signal drift.

In response, continual learning (also known as incremental learning) [18] has emerged as a promising paradigm for real-world BCIs. Continual-learning models are designed to assimilate new information from continuous data streams while preserving and refining previously acquired knowledge, thereby mitigating catastrophic forgetting. In the deep learning literature, van de Ven et al. [19] categorize continual learning into three primary scenarios: (i) task-incremental learning, where task identities are known and a multi-headed output is used; (ii) domain-incremental learning, where the task remains fixed but the input distribution shifts over time; and (iii) class-incremental learning, where new classes are introduced sequentially and the model must retain performance on earlier classes. For MI-BCIs operating with evolving EEG distributions—such as those induced by subject learning, fatigue, or hardware variability—domain-incremental continual learning is particularly relevant, yet remains underexplored, especially in online, multi-class, and dry-electrode settings. Recent efforts have started to explore domain-/session-incremental [20] learning strategies for MI-EEG across shifting distributions, but these studies are still predominantly evaluated under offline or limited deployment settings, and rarely under the combined constraints of dry electrodes, real-time closed-loop control, and multi-class (four-class) decoding. Therefore, the practical question of whether continual adaptation can reliably reduce the offline-to-online gap for dry-electrode, real-time four-class MI control remains insufficiently answered.

Recent deep learning approaches have reported high offline accuracies for four-class MI–BCI classification—for example, EEGNet [15] and transformer-based models [16] can reach approximately 75–80% or higher on public benchmark datasets under subject-dependent offline evaluation protocols. However, these results do not readily translate to online performance. In real-time settings, accuracy is frequently lower and less stable; in a four-class Cybathlon scenario, for instance, performance deteriorated near competition despite extensive training, suggesting high sensitivity to stress-induced EEG shifts and environmental changes [21]. Reviews in stroke neurorehabilitation similarly point out that clinical translation is still limited by low online accuracy and the fact that many “high” performance reports are based on offline analyses rather than closed-loop control [22]. Although virtually embodied feedback can improve subjective usability and engagement [12], it still typically requires prolonged, user-specific calibration, and robust four-class online control remains highly variable across users.

Importantly, studies that are most comparable to the present work are those that evaluate MI decoding under deployment-relevant constraints—namely closed-loop/online operation, multi-class control (especially four-class), and/or wearable-friendly acquisition (e.g., dry electrodes). Across these settings, reported performance is typically lower than offline benchmarks and exhibits larger variability, highlighting a practical gap between “offline accuracy” and “online reliability”. Moreover, direct numerical comparison across studies can be misleading because electrode type (dry vs. wet), cue/feedback design, class cardinality, and closed-loop timing constraints differ substantially; therefore, comparability should be interpreted primarily in terms of setting realism and adaptation capability rather than peak offline scores alone.

Moreover, most prior MI–BCI studies rely on traditional wet electrodes, which are impractical for large-scale or daily use. Dry-electrode EEG provides a more convenient and user-friendly alternative for real-world systems; however, it also introduces practical challenges such as higher and time-varying contact impedance, increased susceptibility to motion/pressure artifacts, and reduced signal-to-noise ratio, which can exacerbate non-stationarity during prolonged use. Recent reviews of portable dry-electrode EEG systems [23] further highlight that these hardware and contact-related factors remain key barriers to stable, repeatable decoding in real-world deployments. As a result, online multi-class (four-class or more) MI–BCIs with dry electrodes remain challenging and relatively underexplored.

To more explicitly address previously reported works, we position our study against three representative research directions. First, prior AO/VR-based MI-BCI systems primarily emphasize training design and user engagement (e.g., VR feedback and AO + MI paradigms) but typically rely on static decoders and do not explicitly handle online distribution shifts during closed-loop control. Second, recent deep models such as EEGNet and transformer-based architectures have achieved strong performance largely under offline evaluations and controlled protocols, leaving a gap to deployment-relevant online reliability. Third, real-world competitive/clinical reports highlight that four-class MI performance can deteriorate under stress and environmental changes and that offline accuracy often overestimates closed-loop performance. Motivated by these limitations, our work focuses on a realistic setting—dry-electrode acquisition with real-time four-class control—and introduces domain-incremental continual adaptation to reduce the offline-to-online gap under non-stationarity.

In this work, we propose a user-centered AO-in-VR MI-BCI framework that explicitly follows a two-phase learning pipeline, thereby addressing key limitations of related works that are often evaluated offline and/or do not explicitly mitigate dry-EEG non-stationarity during closed-loop operation. First, an immersive AO + MI pre-training stage with dry-electrode EEG is used for offline initialization of a CNN–Transformer hybrid decoder. Second, during closed-loop online operation, a continual-learning-enhanced adaptation stage incrementally updates the model using real-time data and feedback, targeting domain-incremental shifts in MI-related EEG. This rarely explored practical combination—dry-electrode EEG, immersive VR, real-time four-class decoding, and continual learning—enables robust multi-class control under realistic constraints. In a four-class MI paradigm (left hand, right hand, both feet, and rest), the proposed strategy improves online accuracy from 47.4% to 66.2% through continual adaptation, while ERD/ERS analyses further corroborate strengthened sensorimotor engagement under AO + MI. By jointly addressing neural activation, dry-electrode signal constraints, and online non-stationarity, the proposed framework narrows the gap between offline benchmarks and reliable real-time multi-class control, supporting practical deployment in neurorehabilitation and assistive BCI applications.

The remainder of this paper is organized as follows. Section 2 reviews relevant literature and theoretical background on MI-BCI, AO + MI paradigms, dry-electrode EEG, and continual learning. Section 3 describes the proposed system architecture, including the AO-in-VR training environment, experimental protocol, two-phase (offline + online) training pipeline, CNN–Transformer hybrid model, baseline methods, and ERD/ERS analysis. Section 4 presents the experimental results, covering online classification performance and neurophysiological findings, followed by an in-depth discussion. Finally, Section 5 concludes the study and outlines future directions for extending the proposed framework to broader real-world BCI and neuroadaptive applications.

## 2. Materials and Methods

### 2.1. Participants and EEG Acquisition

Five healthy, right-handed male participants (aged 20–22 years) were recruited for this study. None reported any history of neurological or psychiatric disorders. All procedures were approved by the local ethics committee, and written informed consent was obtained in accordance with institutional guidelines.

EEG signals were collected using a wireless dry-electrode EEG system (InMex; WellFulfill Ltd., Taoyuan, Taiwan), featuring eight measurement channels, a 24-bit analog-to-digital converter (ADC), and a 1 kHz sampling rate. The system includes integrated Bluetooth and hardware trigger interfaces. EEG signals were transmitted via Bluetooth and acquired in real time using a custom-developed backend built with Python 3.0, which provided a graphical user interface (GUI) for continuous signal monitoring and control.

**Figure 1:**
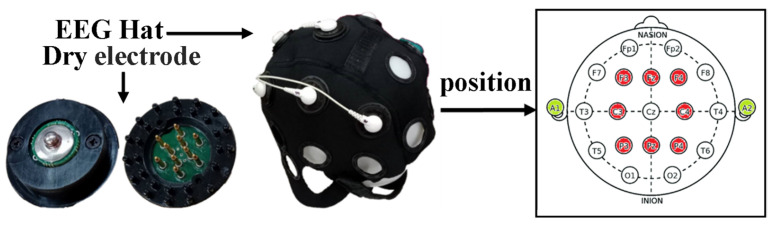
EEG hardware setup with eight biocompatible dry electrodes mounted on a 19-channel cap. Electrode placement followed the 10–20 system, targeting sensorimotor areas; ground and reference were located at the mastoids.

Electrode positioning followed the international 10–20 system. Eight active electrodes were placed over key sensorimotor regions: F3, F4, C3, Cz, C4, P3, Pz, and P4. Ground and reference electrodes were located at the left and right mastoids, respectively. The electrode layout is shown in Figure 1.

### 2.2. System Architecture and Experimental Setup

**Figure 2:**
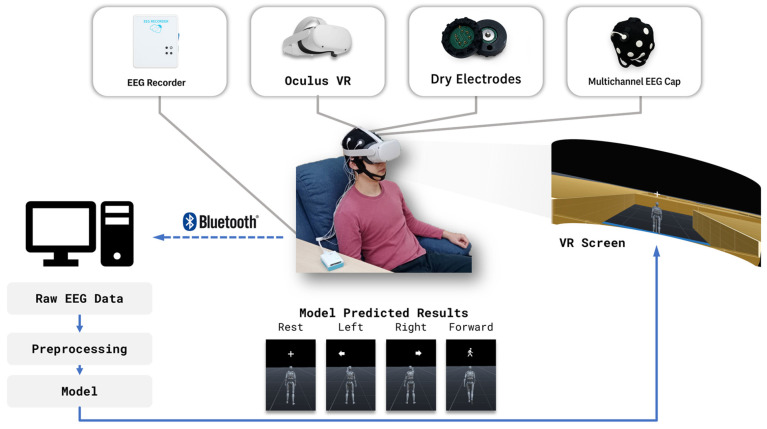
Overview of the system architecture. EEG signals recorded via a dry-electrode cap were transmitted to a PC, preprocessed, and classified by a pre-trained model. Predicted motor imagery outputs were sent to a Unity-based VR environment to control avatar navigation.

As illustrated in Figure 2, participants were seated in a fixed-position ergonomic chair to minimize body movement during the experiment. Each participant wore a dry-electrode EEG cap and a VR headset. Prior to each session, electrode positions were calibrated and adjusted to ensure optimal contact and signal quality.

EEG data were continuously recorded from eight channels using a wireless EEG acquisition system. Raw EEG data were transmitted in real time to a host computer via Bluetooth. Upon reception, the signals were band-pass filtered between 8 and 30 Hz using a third-order Butterworth infinite impulse response (IIR) filter to retain relevant sensorimotor rhythms.

The filtered EEG data were segmented using a 2 s sliding window with a step size of 0.1 s, resulting in an overlap of 1.9 s between consecutive segments. Each EEG segment was input into a deep learning-based classification model to identify one of four motor imagery (MI) states: left turn, right turn, forward movement, or idle. The classification output was then transmitted to a Unity-based VR environment, where the corresponding avatar action was rendered in real time based on the predicted MI state.

### 2.3. Model Framework and Training Strategy

The proposed experimental model integrates Action Observation (AO) and Motor Imagery (MI) strategies to construct a personalized, pre-trained BCI classification model for each participant. Because dry-electrode EEG is prone to time-varying non-stationarity (e.g., contact changes, motion artifacts, fatigue), to adapt to the dynamic changes in the user’s brain state, a continual learning mechanism is incorporated to enable automatic updates of model parameters over time. This approach aims to sustain and progressively enhance MI classification accuracy in real-world applications. All update operations are performed within the same fixed task and class set, i.e., a domain-incremental setting.

Consistent with the framework outlined in Section 1, the proposed MI-BCI system follows a two-phase training pipeline: (i) an offline AO + MI pre-training phase using dry-electrode EEG to initialize a CNN–Transformer hybrid decoder, and (ii) an online continual-learning phase, in which the pre-trained model is incrementally adapted based on real-time feedback data. The goal is to implement a domain-incremental continual learning scheme that tracks gradual distribution shifts in MI-related dry-EEG while preserving previously acquired knowledge. In this domain-incremental scenario, the MI classes remain unchanged across sessions, whereas the input distribution may drift over time.

The overall system architecture consists of the following components.

#### 2.3.1. Pre-Training Phase (Offline Training)

In the offline pre-training phase, participants performed AO + MI and pure MI tasks while wearing the VR headset, so that all visual stimuli (fixation cross, directional cues, and avatar movements) were presented in an immersive virtual environment. Following the experimental paradigm described in [24], each participant underwent three consecutive days of training, completing five experimental runs per day (15 runs in total). Each run consisted of randomized trials involving three types of motor imagery (MI) tasks: left hand, right hand, and both feet. Each MI type was presented in three sets, and each set included three trials: two Action Observation + Motor Imagery (AO + MI) trials followed by one pure MI trial.

**Figure 3:**
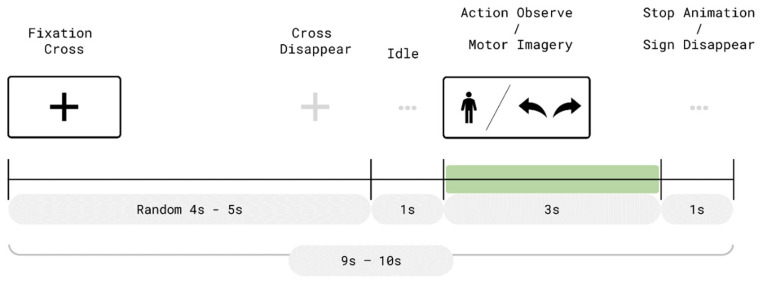
Trial timeline during the offline AO + MI pre-training phase. Each 9–10 s trial in the offline sessions comprised a fixation period (4–5 s), a 1 s preparatory interval, a 3 s AO + MI or pure MI phase with visual cues, and a 1 s post-trial interval.

As illustrated in Figure 3, each trial consisted of two time periods: a resting period (randomly lasting 4–5 s) and a 3 s movement period. At the beginning of each trial, a fixation cross was presented at the center of the VR display during the resting phase. After the fixation period, a 1 s preparatory interval was given before entering the movement phase.

**Figure 4:**
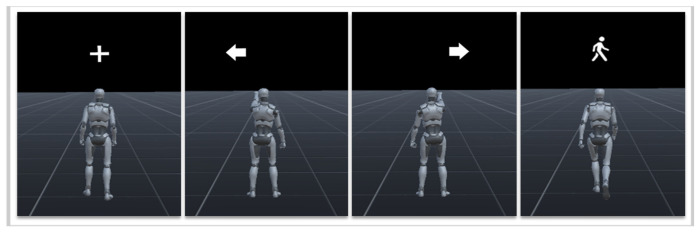
Visual interface for Action Observation + Motor Imagery (AO + MI) trials in the VR environment during the offline training phase. In these offline pre-training sessions, the avatar performs one of four actions corresponding to the MI classes: rest (fixation cross), left turn (left arrow), forward movement (walking icon), and right turn (right arrow).

In AO + MI trials, a virtual avatar in the VR environment performed the designated movement while a corresponding directional cue was displayed. For example, in a right-hand trial, the avatar raised its right arm and a right-pointing arrow appeared above its right shoulder, as shown in Figure 4. Participants were instructed to simultaneously imagine performing the same movement.

**Figure 5:**
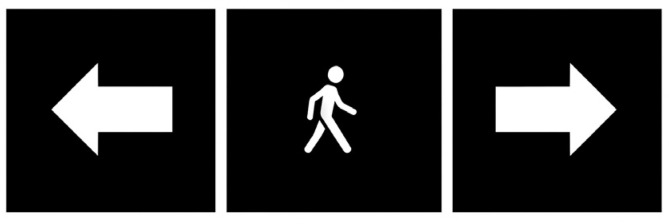
Directional visual cues used during pure motor imagery (MI) trials in the offline training phase. In these offline pre-training sessions, participants were guided by three visual symbols: left arrow, walking icon (forward movement), and right arrow.

In contrast, during pure MI trials (Figure 5), the avatar was not shown. Only the directional cue appeared within the VR scene, prompting the participant to perform motor imagery without any concurrent visual movement observation. Each movement phase lasted 3 s. Afterward, the avatar ceased movement (if present) and the directional cue disappeared. A 1 s interval followed before the next trial began with the reappearance of the fixation cross. Each run lasted approximately 4.5 min, and each movement class was presented in a total of 27 trials across the session.

For model training, EEG segments corresponding to the three MI tasks and the inter-trial resting periods were labeled as four classes: left-hand MI, right-hand MI, both-feet MI, and rest. During the subsequent online phase, these three MI classes were mapped to left-turn, right-turn, and forward navigation commands, respectively, while the rest class served as the idle state (see Section 2.3.2).

#### 2.3.2. Online Continual-Learning

**Figure 6:**
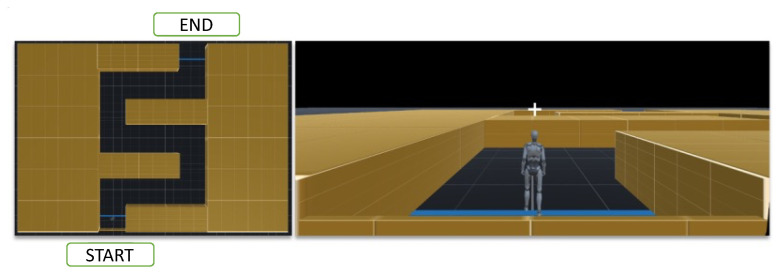
Serpentine maze and third-person VR view used for motor imagery-based navigation during the online continual-learning phase. In these online sessions, participants controlled the avatar using four-class MI commands while the pre-trained decoder was incrementally updated with feedback data.

As shown in Figure 6, the online continual-learning phase involved real-time navigation through a serpentine VR maze using motor imagery (MI). Participants controlled a virtual avatar from a fixed starting point to the goal by responding to directional cues—left turn, right turn, or forward movement—with the corresponding imagined movement. During each imagery period, EEG signals were classified by the pre-trained model. If the predicted class matched the instructed direction, the avatar executed the intended movement; otherwise, the avatar remained stationary, and the trial was repeated until successful classification.

This phase was designed as a domain-incremental continual learning extension of the pre-training protocol, enabling further refinement of the decoder under realistic, closed-loop conditions. Each feedback session was treated as a new domain in a domain-incremental continual learning scenario: the model was initialized from the offline pre-trained weights and then incrementally updated with data collected in the current and previous sessions, instead of being retrained from scratch. By progressively fine-tuning the model on accumulated feedback trials, the system aimed to track session-to-session shifts in the dry-EEG distribution while retaining prior knowledge, thus mitigating catastrophic forgetting. The online continual-learning experiment was conducted over three consecutive days, with three sessions per day, resulting in a total of nine adaptation runs per participant.

To minimize environmental interference and preserve consistency with the pre-training phase, participants remained seated and followed the same posture and trial timing structure. This design ensured that EEG variability was primarily attributed to neural adaptation rather than physical artifacts.

**Figure 7:**
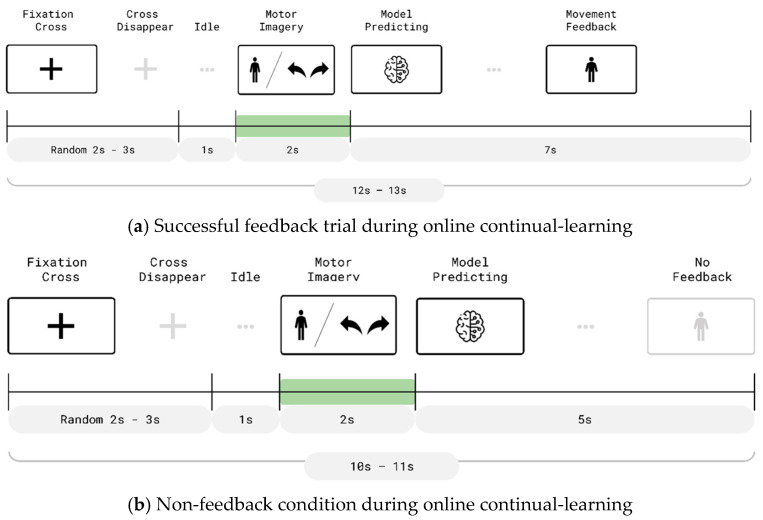
Timelines of online trials during the continual-learning phase, with and without real-time feedback. (**a**) Feedback trial (12–13 s duration), consisting of a 2–3 s fixation period, 1 s idle period, a 2 s MI phase, followed by model prediction and a 7 s movement feedback phase. (**b**) No-feedback trial (10–11 s duration), which omits the feedback phase. After the MI phase and model prediction, the trial proceeds directly to the next cycle while the decoder continues to be updated within the online continual-learning framework.

The trial timeline for the online continual-learning phase is illustrated in Figure 7a,b. At the beginning of each trial, a fixation cross was displayed for a randomly selected duration of 2–3 s, followed by a 1 s preparatory interval. A directional cue (e.g., left turn, right turn, or forward) then appeared on the VR display for 2 s, during which participants were instructed to perform the corresponding MI task.

Immediately afterward, the system entered a 4 s classification window, continuously analyzing EEG data in real time. If the predicted class matched the presented cue, the avatar executed the intended movement and visual feedback was displayed (see Figure 7a). Otherwise, the avatar remained stationary and the trial was repeated following a 1 s preparatory interval (Figure 7b). To minimize participant fatigue and mitigate performance anxiety resulting from repeated failed attempts, the system limited the number of retries per instruction to three. If all three attempts failed, the system force-passed the trial and proceeded to the next command.

For real-time classification, a 2 s sliding window was employed with a 1.9 s overlap, yielding a new prediction every 0.1 s. To improve output stability, a temporal smoothing mechanism was applied: five consecutive identical predictions were required within the MI period to confirm a valid classification. If this consistency criterion was not met, the model output defaulted to a “rest” state. While this method enhanced classification stability, it introduced an approximate 0.5 s response latency.

### 2.4. EEG Conformer Model Architecture

The proposed decoder adopts a lightweight CNN–Transformer hybrid design to balance representational power and real-time feasibility. The CNN front-end serves as an efficient local feature extractor that captures short-range temporal dynamics and spatially distributed sensorimotor patterns (e.g., ERD/ERS-related signatures) with low computational overhead, which is desirable for dry-electrode EEG, where signal quality is noisier and latency constraints exist. However, convolution alone can be limited in modeling longer-range temporal dependencies and global inter-channel relationships that may shift across sessions. Therefore, a Transformer encoder is introduced to learn context-aware representations via attention, enabling the model to better integrate distributed evidence across time and channels under non-stationary conditions. A qualitative, implementation-oriented comparison of practical considerations across representative architectures (EEGNet, S3T, and the proposed model) is summarized in Supplementary Table S1.

**Figure 8:**
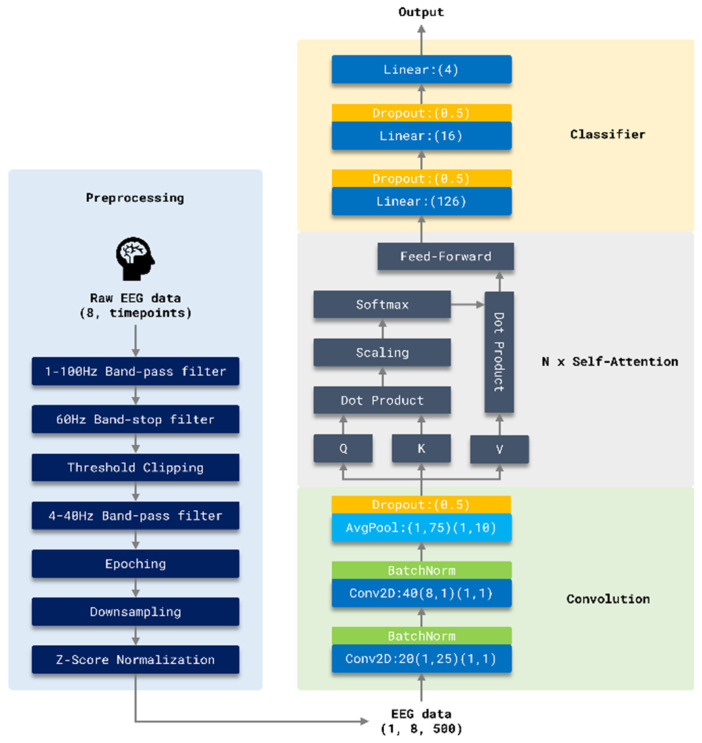
End-to-end architecture of the proposed EEG classification model. The system comprises four main stages. (1) Preprocessing: Raw 8-channel EEG signals undergo multi-band filtering, threshold clipping, epoch segmentation, downsampling, and Z-score normalization. (2) Convolution: Two 2D convolutional layers with batch normalization, average pooling, and dropout are applied to extract local spatiotemporal features. (3) N × Self-attention: A multi-head attention mechanism with feed-forward layers captures long-range temporal dependencies and integrates global contextual information. (4) Classifier: A fully connected network with dropout produces the final four-class classification output.

Figure 8 illustrates the architecture of the proposed model, which consists of two primary components: the preprocessing pipeline and the core classification model.

#### 2.4.1. Preprocessing Pipeline

The preprocessing pipeline was designed to convert raw EEG signals into a structured format suitable for classification. This process consisted of a series of steps: band-pass filtering, artifact suppression, epoch extraction, downsampling, and normalization.

Initially, a band-pass filter (1–100 Hz) was applied to isolate the relevant EEG frequency components, and a 60 Hz band-stop filter was used to eliminate power line interference. To suppress transient artifacts caused by body movement or cable tension, the signal amplitudes were clipped to a range of ±50 µV (i.e., ±5 × 10^−5^ V).

Following filtering and clipping, the signal was further restricted to the 4–40 Hz band to emphasize motor-related activity. Event triggers were then used to segment the continuous EEG stream into 2 s epochs, each comprising 2000 time points at the original sampling rate of 1000 Hz.

To reduce computational load, the epochs were downsampled to 250 Hz, yielding 500 samples per segment. Each channel was subsequently normalized using Z-score normalization, defined as:

(1) $$x_{o}=\frac{x_{i}-\mu }{\sqrt{\sigma^{2}}}$$

where $x_{o}$ is the normalized signal, $x_{i}$ is the signal after filtering and downsampling, and μ and $\sigma^{2}$ denote the mean and standard deviation calculated over the training dataset. This normalization step reduced inter-channel variability and mitigated nonstationarity, enhancing model stability and generalization.

After preprocessing, the raw EEG data originally shaped as 8 × samples (eight channels) were converted into a 3D tensor of dimensions trials × 8 × 500, which was used as input for subsequent model training and classification.

#### 2.4.2. Core Classification Model (CNN–Transformer Hybrid/EEG Conformer)

The core classification model adopts a CNN–Transformer hybrid architecture based on the EEG Conformer proposed by Song et al. [25]. It comprises three main modules: a convolutional module, a self-attention module, and a classifier module. The convolutional and self-attention components together serve as a feature extractor, capturing both local and global patterns in the EEG signals. The resulting feature vectors are subsequently passed to the classifier module to generate four-class motor imagery (MI) predictions. For reproducibility, the complete architecture hyperparameters derived from Figure 8 and the released implementation (including the Transformer configuration, e.g., N = 3 encoder blocks, h = 10 heads, d_model = 40, FFN hidden dim = 160, and dropout *p* = 0.5) are summarized in Supplementary Table S2.

The convolutional module consists of two sequential convolutional layers designed to extract temporal and spatial features. The first layer applies 20 kernels of size 1 × 25 with a stride of 1 × 1 to model temporal dependencies within each channel. The second layer uses 40 kernels of size 8 × 1 (stride 1 × 1) to capture inter-channel spatial relationships across the eight input channels. This hierarchical design enables the model to learn a robust set of local representations across time and space.

Each convolutional layer is followed by batch normalization and an exponential linear unit (ELU) activation function to accelerate convergence and reduce overfitting. To further lower computational complexity, an average pooling layer with a kernel size of 1 × 75 and a stride of 1 × 10 is applied, performing temporal downsampling and dimensionality reduction. The resulting output is reshaped into a matrix of shape (samples × number of convolutional filters), which serves as input to the subsequent self-attention module.

The self-attention module is designed to capture long-range temporal dependencies. Input features are linearly projected into query (Q), key (K), and value (V) matrices. Attention weights are computed via scaled dot-product attention, where the dot product of *Q* and *K* is scaled by the square root of the key dimension, followed by a softmax activation to yield normalized attention scores. These weights are applied to the *V* matrix to produce a context vector:

(2) $$Attention(Q,K,V)=Softmax\left(\frac{QK^{T}}{\sqrt{k}}\right)V$$

The attention output is then passed through a two-layer feed-forward network to further enhance feature expressiveness. This self-attention block is repeated N times to increase temporal modeling depth.

To enhance representational diversity, a multi-head attention mechanism is employed. Q, K, and V matrices are divided into h attention heads, each processed independently and subsequently concatenated. Here l denotes the head index (i.e., the l-th attention head), where l ∈ {0, 1, …, h − 1}, and h is the total number of heads. For each head, the projected query, key, and value matrices ($Q_{l}$, $K_{l}$, $V_{l}$) are obtained by applying head-specific linear projections to Q, K, and V, respectively. The output of each head is computed via scaled dot-product attention, and the final MHA output is formed by concatenating all head outputs (followed by an output projection, if applicable):

(3) $$MHA\left(Q,K,V\right)=\left[head_{0};\ldots ;head_{h-1}\right],\;{head}_{l}=Attention(Q_{l},K_{l},V_{l})$$

Finally, the classifier module consists of two fully connected layers. The first layer includes 126 units with ELU activation, followed by a second layer with 16 units and a four-class softmax output corresponding to the MI categories. Dropout regularization is applied between layers to mitigate overfitting and improve generalization.

### 2.5. Model Training and Optimization Pipeline

**Figure 9:**
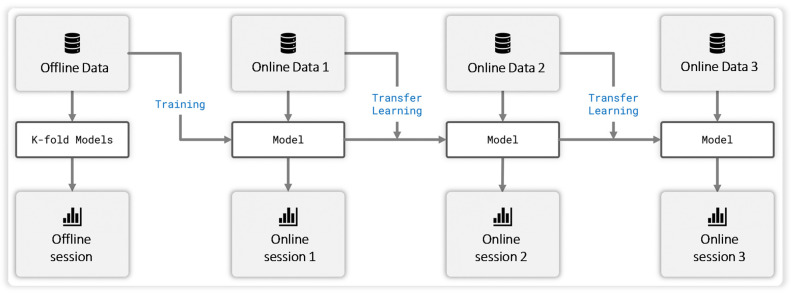
Training flow for the offline pre-training and online continual-learning phases.

During the pre-training data processing phase, stratified 5-fold cross-validation was initially performed to assess the generalization performance of the model. Following this evaluation, the entire dataset was used to train the final version of the model, which was subsequently deployed in the online feedback experiments. Stratification ensured that each fold preserved the same class distribution as the original dataset, thereby improving the reliability of performance estimation across motor imagery (MI) categories. The overall training procedure—offline AO + MI pre-training to initialize the CNN–Transformer decoder, followed by progressive fine-tuning across three online feedback sessions (Online session 1–Online session 3)—is summarized in Figure 9, which illustrates the offline–online training and domain-incremental continual-learning pipeline used in this study.

Unlike standard cross-validation schemes, the dataset in this study consisted of two trial types: Action Observation (AO) and Motor Imagery (MI). Since both the experimental focus and real-time feedback control were exclusively based on MI, a custom partitioning strategy was employed. Specifically, only the MI data were partitioned into five folds for training and testing. All AO trials were incorporated into the training set of each fold, ensuring that the model had full access to AO data during training, while the test set consisted exclusively of MI trials. This partitioning strategy ensured that the validation setting more accurately reflected the conditions encountered during the online MI-based feedback phase.

The original dataset comprised 90 AO and 45 MI trials, yielding a 2:1 ratio. After partitioning, each fold contained 126 training samples and 9 testing samples. To enhance model robustness, data augmentation was applied to both training and test sets after the folds were defined to ensure independence. For each movement class (left hand, right hand, both feet), five overlapping 2 s EEG segments were extracted from the event trigger point at 0.1 s intervals. For the rest class, segments were extracted starting 3 s before the event. Because rest trials were more abundant, random subsampling was applied post-augmentation to ensure class balance. This process expanded each class to 675 trials. Performing augmentation only after data partitioning avoided sample overlap between training and testing, thereby preserving statistical independence.

During the online continual-learning phase, each participant completed three adaptation sessions, during which the model underwent two rounds of fine-tuning. A pre-trained model was initially developed using all available offline data and subsequently updated after the first and second sessions based on the successfully triggered trials collected in each session. The fine-tuning process involved adjustments to key hyperparameters—such as learning rate, batch size, and number of epochs—to optimize model performance and enhance real-time feedback accuracy. This progressive fine-tuning procedure corresponds to a domain-incremental continual learning strategy: newly collected feedback trials from each session are combined with previously acquired data to update the model, allowing it to adapt to evolving dry-EEG statistics while preserving earlier knowledge learned during offline AO + MI pre-training.

Two data handling strategies were employed based on the outcome of each triggering attempt. In successful trials, where the model accurately predicted the intended movement and the avatar responded accordingly in the VR environment, the movement onset was aligned with the classifier’s decision derived from the sliding window. The motor imagery (MI) trigger point was defined as 2.5 s prior to this motion onset. From that point, five overlapping 2 s EEG segments were extracted at 0.1 s intervals to augment the dataset. Rest-class data were processed using the same approach as in the pre-training phase, with five segments extracted from 3 s before the event trigger.

In contrast, for unsuccessful trials where the model failed to trigger the correct movement after three consecutive attempts, it was assumed that the participant initiated imagery upon cue presentation. In such cases, the cue onset was treated as the MI trigger point, and five 2 s segments were extracted using the same sliding-window method. These augmented samples were incorporated into the training set to ensure that the model could learn from both successful and unsuccessful control attempts.

Before each fine-tuning iteration, the updated dataset was evaluated using the existing model to assess current performance. The data were then split into training and testing sets with an 8:2 ratio. All data augmentation was performed after this split to preserve the independence of the test set, following the same precautions as in offline training.

Pilot runs and optimizer settings. Model development and training were implemented using PyTorch 1.8.1 and executed on an NVIDIA GeForce RTX 2060 GPU (12 GB). A fixed random seed was applied across all runs to ensure reproducibility. We optimized the cross-entropy loss using Adam with β1=0.5, β2=0.999, and weight decay = 1 ×$10^{-4}$. These hyperparameters were selected based on established practice and pilot validation to balance convergence stability and robustness under noisy dry-electrode EEG. Specifically, β2= 0.999 provides a stable second-moment estimate, while the smaller β1=0.5 makes the first-moment (momentum) update more responsive to distribution shifts and trial-to-trial variability, which is particularly relevant during closed-loop online adaptation. The weight decay term acts as L2 regularization to mitigate overfitting given the limited sample size and model capacity.

To make the pilot-run procedure explicit, we conducted pilot runs within the training data using a training-only inner validation split under the offline 5-fold protocol and evaluated validation accuracy and weighted F1 together with convergence stability across folds/runs. The pilot search covered representative choices of learning rate, warmup length/schedule, Adam betas, weight decay, and batch size. For offline pre-training, we adopted a warm-up learning rate schedule (Equation (4)) with $n_{warmup}$ = 10 to stabilize early optimization and reduce instabilities commonly observed in attention-based architectures, using a base learning rate of 3 ×${\;10}^{-4}$ and a total of $n_{epoch}$ = 100 epochs. For online fine-tuning, we used a smaller learning rate 2 × $10^{-5}$ and fewer epochs (20) to reduce overfitting to limited feedback data while meeting real-time constraints (short adaptation horizon and low latency). The detailed hyperparameters for the offline pre-training and online feedback adaptation phases are summarized in Table 1. Representative pilot-run comparisons of optimizer and learning-rate schedule configurations are summarized in Supplementary Table S3.

(4) $$lr\left(epoch\right)=\left\{\begin{array}{c} {lr}_{max}\times \frac{epoch}{n_{warmup}},\;\;epoch\leq n_{warmup}\\ {lr}_{max}\times \frac{n_{epoch}-epoch}{n_{epoch}-n_{warmup}},\;{\;n}_{warmup}<epoch\leq n_{epoch} \end{array}\right.$$

### 2.6. Baseline Models

This section introduces the four baseline models used for pre-training evaluation. The selected models include one Transformer-based architecture—Spatial-Temporal Tiny Transformer (S3T)—and three well-established convolutional neural network (CNN) models: EEGNet, DeepConvNet, and ShallowConvNet.

S3T [26] is a compact model specifically designed for motor imagery (MI) EEG classification, aiming to simultaneously capture spatial and temporal dependencies in EEG signals. The input signals are first transformed using a one-to-many mapping via Common Spatial Pattern (CSP) filtering. The resulting features are stacked and fed into the model. A channel attention mechanism is applied to prioritize task-relevant EEG channels while suppressing irrelevant ones, enhancing the model’s representational precision. After spatial processing, the data are compressed and segmented along the temporal dimension, followed by a temporal attention mechanism to extract global sequential features critical to MI decoding. Final classification is performed via global average pooling and a fully connected layer.

EEGNet [15] is a lightweight end-to-end model for EEG-based classification, composed of three distinct convolutional operations. The first layer is a standard 2D convolution that extracts initial spatial and temporal features. The second is a depthwise convolution, which learns frequency-specific spatial patterns across channels. The third is a pointwise convolution, forming a separable convolution structure that integrates temporal features efficiently. A fully connected layer is used at the end for classification.

DeepConvNet [27] is a deeper end-to-end CNN architecture tailored for MI EEG classification. It consists of four convolution–max pooling blocks. The first block includes two convolutional layers that handle raw EEG inputs, extracting low-level temporal and spatial features. The remaining three blocks progressively increase the depth of learned representations, improving the model’s discriminative capability. Final predictions are made through a fully connected output layer.

ShallowConvNet [27] is a simplified variant of DeepConvNet, featuring a lighter architecture composed of two convolutional layers followed by a classifier. While more compact, it also emphasizes the extraction of key spatial and temporal patterns. Functionally, its architecture mimics the Filter Bank Common Spatial Pattern (FBCSP) approach: the early convolution layers serve as substitutes for bandpass and spatial filters, while the later stages apply squaring, average pooling, and a log activation function to estimate log-variance features across trials. Classification is completed via a fully connected layer.

### 2.7. ERD/ERS Analysis

To examine changes in neural activity following training, event-related desynchronization (ERD) and event-related synchronization (ERS) analyses were performed on trials that were correctly classified by the model during each motor imagery feedback (MI-FB) session. An ensemble averaging approach was applied to these successfully triggered trials to assess whether the model reliably identified physiologically relevant EEG patterns and to evaluate potential enhancements in cortical activation after training.

ERD is characterized by a decrease in power within specific frequency bands (e.g., alpha or beta) in response to motor-related events or stimuli, typically indicating cortical activation. In contrast, ERS represents an increase in band power following an event, often associated with cortical deactivation or inhibition.

In the context of motor imagery tasks, desynchronization (ERD) in the alpha (8–14 Hz) and beta bands is commonly observed over the sensorimotor cortex during imagined hand or foot movements. This is typically followed by resynchronization (ERS) after the movement concludes or during short rest intervals.

The ERD/ERS analysis followed the method proposed by Pfurtscheller et al. [28]. EEG signals were first band-pass filtered in the alpha range (8–14 Hz) to isolate motor-related rhythms. The power of the filtered signal was then computed by squaring the signal amplitude, resulting in a trial-wise power estimate denoted as $P\left(class,t\right)$ for each motor imagery class and trial.

To compute the mean band power across trials for each motor-imagery (MI) class, we first extracted the trial-wise band power P(class,n) and then averaged it over all trials within that class, as defined in Equation (5). Here, P(class,n) denotes the band power of the n-th trial from the specified MI class, and $N_{class}$ is the total number of trials belonging to that class. In this study, class ∈ {left hand, right hand, both feet}. The resulting class-wise mean power P(class) was used to characterize MI-related oscillatory dynamics and to provide neurophysiological validation (e.g., ERD/ERS) of the decoding results.

(5) $$P\left(class\right)=\frac{1}{N_{class}}\sum_{n=1}^{N_{class}}P\left(class,n\right)class\in \{left\;hand,right\;hand,both\;feet\}$$

To compute the baseline power reference, we used the rest condition and averaged the power over both time samples within each trial and all rest trials, as shown in Equation (6). The baseline $P_{rest}$ is a scalar constant used as the reference for subsequent ERD/ERS percentage calculations. Here, P(n,s) denotes the time-resolved power in the target frequency band at the s−th sample of the n−th rest trial, $N_{rest}$ is the number of rest trials, and $N_{sample}$ is the number of samples per trial.

(6) $$P_{rest}=\frac{1}{N_{rest}N_{sample}}\sum_{n=1}^{N_{rest}}\sum_{s=1}^{N_{sample}}P(n,s)\;\;$$

Subsequently, the ERD/ERS percentage for each MI class was computed using Equation (7):

(7) $$ERD/ERS\left(class\right)=\frac{P(class)-P_{rest}}{P_{rest}}\times 100\;(\%)$$

This equation quantifies the relative power change between MI trials and the resting-state baseline. Negative values (ERD) indicate power attenuation (desynchronization), reflecting cortical activation associated with motor imagery, whereas positive values (ERS) indicate power enhancement (synchronization), depending on the frequency band of interest.

## 3. Results

### 3.1. Performance Evaluation of Pre-Training and Online Adaptation

To evaluate the performance of the pre-trained model, 5-fold cross-validation was conducted using accuracy and weighted F1 score as the primary evaluation metrics. All baseline models were trained following the procedures described in Section 2.5.

All results reported in this section refer to four-class MI decoding (left hand, right hand, both feet, rest) using 8-channel dry-electrode EEG. Thus, the chance-level accuracy for uniform random guessing is 25%.

Table 2 presents a comparative summary of performance across the proposed model and four baseline models. Averaged across five participants, the proposed model achieved the most stable and superior performance, with a mean accuracy of 52.78% and a weighted F1 score of 52.21%. These scores outperformed all baseline models.

Notably, when compared with the best-performing baseline, ShallowConvNet [27], the proposed model achieved an absolute gain of +3.09 percentage points in accuracy and +2.67 percentage points in weighted F1 score, making it the preferred choice for subsequent online adaptation and parameter update experiments.

Table 3 compares the performance of the pre-training model with that of the model at three stages of online continual-learning phase: Online session 1, Online session 2, and Online session 3. “Pre-training” denotes the results obtained through 5-fold cross-validation on the initial training dataset. “Online session 1” reflects the performance when the pre-trained model was directly applied to the data from the first motor imagery (MI) feedback session without any adaptation. “Online session 2” and “Online session 3” represent the model’s performance after the first and second rounds of fine-tuning, respectively, using newly collected feedback data to iteratively update the model parameters.

For clarity, Table 2 and Table 3 report both subject-level results and the group average, and we explicitly summarize the key trends (best baseline gap and online gains) in the text to avoid over-reliance on dense tables.

As summarized in Table 3, the model exhibits a typical offline-to-online drop when directly deployed without adaptation (Online session 1 vs. Pre-training), followed by progressive recovery and improvement after continual fine-tuning. Specifically, mean accuracy/weighted F1 changed from 52.78%/52.21% (Pre-training) to 47.42%/46.83% (Online session 1), then to 52.77%/49.71% (Online session 2), and finally to 66.20%/64.50% (Online session 3), corresponding to a net gain of +18.78 percentage points in accuracy and +17.67 percentage points in weighted F1 from Online session 1 to Online session 3. These trends highlight the benefit of continual adaptation under dry-electrode closed-loop conditions.

Notably, the final online performance in Online session 3 (66.20% mean accuracy) exceeded the offline cross-validation accuracy (52.78%), indicating that continual adaptation can not only recover but surpass the initial offline benchmark.

### 3.2. Feature Representation Visualization Using t-SNE

**Figure 10:**
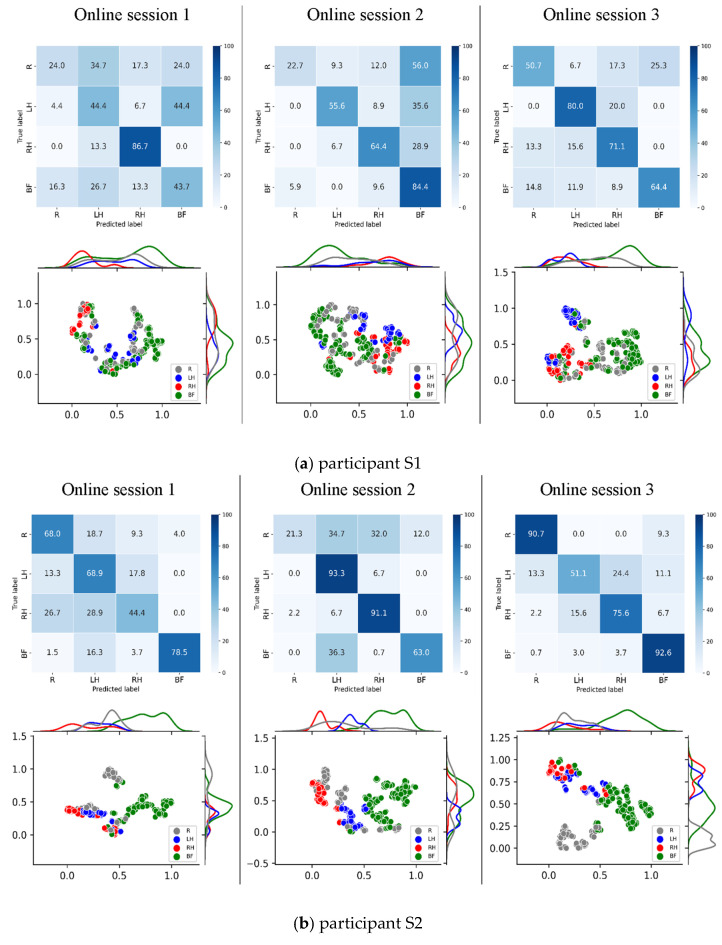
Row-normalized confusion matrices and corresponding t-SNE projections for participants S1 (left) and S2 (right) after model adaptation across three feedback sessions (Online session 1–Online session 3). Confusion matrices are row-normalized to facilitate interpretation of class-wise errors. Diagonal concentration becomes clearer with adaptation for some participants (e.g., S2), whereas residual off-diagonal confusions remain non-negligible for more challenging participants (e.g., S1), consistent with their session-wise performance. The t-SNE plots are presented as qualitative, supportive visualizations of the learned latent features and are interpreted together with the quantitative closed-loop results in Table 3.

To further examine changes in internal feature representations before and after model adaptation, t-distributed stochastic neighbor embedding (t-SNE) [29] was applied to visualize the output feature vectors from the self-attention module of the proposed CNN–Transformer (EEG Conformer) model in a two-dimensional space. Figure 10a,b present the row-normalized confusion matrices and the corresponding t-SNE scatter plots for participants S1 and S2 after model adaptation. While adaptation tends to increase class separability, the effect is heterogeneous across subjects: S2 shows a clearer diagonal concentration and reduced off-diagonal confusions, whereas S1 retains comparatively lower diagonal values with non-negligible residual confusions, consistent with its lower session-wise accuracy. Therefore, Figure 10 is interpreted as supportive, qualitative visualization, with the primary evidence provided by the quantitative results in Table 3.

Specifically, motor imagery classes with higher classification accuracy—reflected by darker diagonal cells in the confusion matrix—tend to exhibit more compact intra-class clusters and clearer inter-class boundaries in the t-SNE plots. Conversely, classes with lower performance tend to show greater dispersion and overlap, indicating increased ambiguity in feature encoding.

This qualitative concordance is presented as a supportive interpretation of the representation structure and is discussed together with the quantitative results in Table 3.

### 3.3. Progressive Feature Representation and Neurophysiological Analysis via ERD/ERS

With successive rounds of online adaptation, the t-SNE projections for representative participants (S1 and S2) qualitatively showed more compact within-class groupings and clearer between-class separation in the 2-D embedding space. Notably, MI classes associated with lower decoding accuracy still exhibited overlap and less distinct clusters, which is consistent with the corresponding misclassification patterns in the confusion matrices.

To further examine the neurophysiological basis of MI-related performance, we analyzed event-related desynchronization/synchronization (ERD/ERS) under Action Observation (AO) and Motor Imagery (MI), following Pfurtscheller et al. [28]. For each trial, the 3 s interval after cue onset was defined as the MI window, and the 2 s pre-cue interval served as the resting baseline. ERD/ERS was computed for left-hand (LH), right-hand (RH), and both-feet (BF) imagery using sensorimotor channels (C3, Cz, and C4). Table 4 summarizes ERD/ERS statistics across participants. Overall, AO elicited stronger desynchronization (larger-magnitude ERD) than MI in several conditions, suggesting enhanced sensorimotor engagement during AO priming. Wilcoxon signed-rank tests indicated significant AO–MI differences in selected channel–task pairs (*p* < 0.05). These findings support AO as an effective priming mechanism that strengthens SMR modulation and provides informative AO + MI data for initializing the offline decoder in our two-phase framework.

We note that ERD/ERS changes are not uniform across subjects; in particular, left-hand imagery shows larger inter-subject dispersion (Table 4a), and therefore group-level trends should be interpreted together with the reported subject-level variability rather than as consistent gains for all participants.

### 3.4. Neurophysiological Validation of Model Predictions During Online Adaptation

To assess the physiological plausibility and interpretability of the model’s predictions during online adaptation, we performed an ERD/ERS-based analysis specifically on trials that were correctly classified as successful triggers. For each trial, a 2.5 s interval before the model-triggered movement was designated as the MI window, assuming participants began MI preparation prior to the system response. The 2 s period prior to cue onset served as the baseline for ERD calculation.

The frequency band of interest (alpha) and electrode selections (C3, Cz, C4) were consistent with those used in the pre-training ERD/ERS analysis. ERD values were calculated accordingly, and the results across all five participants are presented in Table 5.

Overall, trials identified as correctly triggered by the model were associated with EEG patterns indicative of motor imagery. The mean ERD values showed a progressive increase from Online session 1 to Online session 3, suggesting strengthened neural engagement with repeated real-time training.

Specifically, for the left-hand imagery class, the C4 channel exhibited a statistically significant increase in ERD value in Online session 3 compared to Online session 1, as confirmed by a Wilcoxon signed-rank test (*p* < 0.05). Although other comparisons did not reach statistical significance, a consistent trend was observed: ERD values in Online session 3 were generally higher than those in Online session 1 across most channels and classes.

Together with the behavioral improvements in four-class online control, these findings reinforce the conclusion that the proposed continual-learning framework progressively captured more functionally meaningful neural representations, providing neurophysiological validation for the adaptive decoding strategy.

Similarly, during online adaptation (Table 5), ERD values exhibit substantial subject- and class-dependent variability; thus, we focus on the overall directional trend (Session 3 vs. Session 1) while explicitly reporting dispersion and statistical evidence for the key comparisons rather than implying uniform improvements across all channels/classes.

To reduce redundancy and improve readability, we will merge the dense subject-level ERD/ERS tables into a compact summary in the main text (group Mean ± SD, Δ, and statistical results), while retaining the full subject-level tables as Supplementary Materials for transparency and reproducibility.

## 4. Discussion

### 4.1. Addressing Limitations of Conventional MI-BCI Systems Through Action Observation

Conventional motor imagery-based brain–computer interface (MI–BCI) systems and datasets typically rely on simple visual or auditory cues to prompt motor imagery execution. However, such abstract and minimal instructions often lack concrete and intuitive movement guidance, making it difficult for users—especially those new to BCI—to understand how to effectively generate motor imagery. This can lead to increased cognitive load and reduced training efficiency. Moreover, in the early stages of training, when the classification model has not yet been established, the system is unable to provide real-time feedback. This absence of feedback can result in frustration, reduced motivation, and lower engagement among users. Critically, the lack of action-specific clarity and consistent feedback can also contribute to variability in EEG patterns across sessions, thereby undermining model stability and generalization.

To address these challenges, the present study employed action observation (AO) to assist motor imagery (MI), offering participants concrete and dynamic visual references to enhance the vividness and interpretability of the imagery task [30]. In the AO + MI experiments, we observed clear event-related desynchronization (ERD) in sensorimotor regions associated with right-hand and both-feet imagery, indicating that AO-augmented MI effectively activates the corresponding cortical areas. However, the effectiveness of AO appeared to vary across motor classes. Specifically, for left-hand imagery, AO + MI alone was insufficient to elicit stable and robust ERD responses. This class-dependent variability may be related to factors such as hand dominance, individual imagery strategy, and the limited dry-electrode montage, and therefore should not be interpreted as an inherent limitation of AO itself. These observations motivate the need for personalized adaptation to stabilize class-specific representations during closed-loop use. Consistent desynchronization in the relevant cortical areas only emerged after additional training through an online continual-learning phase, during which the system provided real-time feedback and model updates based on the user’s imagery performance. These findings suggest that while AO serves as a useful priming tool for MI–BCI training, motor-specific adaptation via online learning remains essential for achieving reliable cortical engagement, particularly for non-dominant or less frequently imagined movements. In our framework, AO-primed offline training in an immersive VR environment provides a more consistent initial representation of MI-related activity, which is then refined during the online continual-learning phase. By coupling AO + MI pre-training with closed-loop feedback on dry-electrode signals, the system directly addresses the lack of intuitive guidance and unstable online performance frequently reported in conventional four-class MI–BCIs.

### 4.2. Neurophysiological Implications of AO + MI and Training Effects Across Sessions

As summarized in Table 4, action observation (AO) consistently elicited stronger event-related desynchronization (ERD) responses than pure motor imagery (MI), particularly in the C3 and Cz channels during left-hand imagery and in the C3 channel during both-feet imagery. These differences reached statistical significance (*p* < 0.05, Wilcoxon signed-rank test) and align with prior findings [10], which indicates that AO + MI strategies can significantly enhance cortical activation in sensorimotor regions.

In addition to its neurophysiological benefits, AO + MI also helps novice users more easily engage in motor imagery [31], serving as an intuitive scaffold for acquiring MI skills. Notably, mirror neuron activation elicited by AO is highly resilient to repetition, showing minimal fatigue effects even with frequent stimulation [32], making AO especially suitable for long-term BCI training protocols.

While most prior studies adopt either AO or MI in isolation, our method uniquely integrates alternating AO and MI phases, where participants recall the observed AO movement during the subsequent MI task. This reinforced mental representation facilitates more vivid and consistent MI execution, and allows the offline-trained model to capture stronger and more stable ERD features, thereby laying a solid foundation for reliable online adaptation. From an algorithmic perspective, these AO-induced ERD patterns provide informative training examples for the CNN–Transformer decoder, strengthening its initial feature space before domain-incremental adaptation. This is particularly important in dry-electrode settings, where signal-to-noise ratios are lower and robust initialization can substantially reduce the burden on subsequent continual learning.

The importance of real-time BCI feedback in enhancing user control within immersive environments was also evident in this study. Each participant completed three VR-based feedback sessions, each containing three route trials. While trial durations within a single session remained relatively consistent, average task completion time decreased across sessions, indicating that participants were able to navigate the same virtual paths more efficiently in later sessions.

The across-session reduction in VR task completion time suggests improved closed-loop controllability with practice. However, given the relatively short training duration per session, we interpret this behavioral trend cautiously and do not claim direct evidence of neuroplasticity. The improvement could reflect a combination of factors, including better familiarity with the VR interface, more consistent imagery strategy execution, and the model’s progressive alignment to the user-specific EEG distribution through continual adaptation. Importantly, this behavioral trend is consistent with the session-wise decoding gains and reduced confusion observed in the online results, while the ERD/ERS analyses provide complementary physiological evidence of sensorimotor engagement during AO priming.

To examine how participants’ EEG representations evolved across online sessions during closed-loop training, we employed t-distributed stochastic neighbor embedding (t-SNE) as a qualitative visualization tool. The purpose of this analysis was to explore whether the continual adaptation process was associated with increased within-class compactness and clearer between-class separation in the learned feature space for representative participants. t-SNE was selected because it provides an intuitive visualization of local neighborhood structures in high-dimensional embeddings produced by the deep model. Our goal was not to benchmark dimensionality-reduction techniques, but rather to obtain an interpretable view of how internal feature representations changed across sessions. Principal component analysis (PCA) was therefore not used as the primary visualization approach because its linear projection may not adequately reveal potentially nonlinear structures in deep EEG feature embeddings. Although alternative nonlinear methods such as UMAP could also be applied, the objective of this analysis was not to compare visualization algorithms, but to use a commonly adopted and widely interpretable technique to complement the quantitative evaluation. Accordingly, the t-SNE plots should be interpreted as qualitative and supportive visualizations rather than definitive evidence of performance improvement. The primary conclusions of this study are supported by the quantitative closed-loop results, including classification accuracy, weighted F1-score, row-normalized confusion matrices, and neurophysiological analyses. In our implementation, PCA was used only for initialization of the t-SNE embedding, and the perplexity parameter was empirically set to 20 to ensure stable and reproducible visualization under a fixed random seed.

In our implementation, AO stimuli were presented from a third-person perspective, featuring a robot-like virtual avatar. While first-person perspectives are generally considered to promote stronger subjective agency [33], the observed ERD magnitudes induced by third-person AO remained significantly higher than those from pure MI. This suggests that even without first-person visual feedback during MI, the third-person AO still provided sufficient visual context and functional embodiment to enhance cortical activation.

### 4.3. Model Architecture Analysis and Training Progression

Beyond the neurophysiological findings, the proposed system can also be viewed as a domain-incremental continual-learning engine operating on dry-electrode, four-class MI data in real time. From an expert-systems perspective, this corresponds to an adaptive inference module that continuously updates its internal representation as new evidence (feedback trials) becomes available.

The proposed hybrid CNN–Transformer architecture, inspired by the design introduced by Song et al. [25], demonstrated superior classification performance compared to traditional CNN models and CSP-based Transformer structures, as summarized in Table 2. A complementary, implementation-oriented qualitative summary of practical considerations (e.g., training stability, overfitting tendency, computational/maintenance overhead, and online suitability) across representative architectures (EEGNet, S3T, and the proposed model) is provided in Supplementary Table S1. In this model, the convolutional module automatically extracted low-level temporal and spatial features from EEG signals, while the self-attention module captured long-range temporal dependencies and facilitated global feature integration. This configuration outperformed models that relied solely on localized CNN filters.

While manually engineered features such as common spatial patterns (CSPs) have proven effective in extracting task-relevant cortical information [14], the S3T model [26], which integrates CSP with Transformer layers, lacked hierarchical representation learning for localized signal variations. As a result, its generalization capability was inferior to the end-to-end learning pipeline adopted in this study.

However, a potential limitation of the proposed architecture lies in the choice of convolutional kernel sizes. Smaller kernels are advantageous for capturing fine-grained features but may amplify noise, whereas larger kernels offer broader context at the cost of reduced sensitivity to subtle signal fluctuations. Future research may explore adaptive kernel sizing strategies to better balance temporal resolution and robustness in EEG decoding.

Across the offline and online evaluations (Table 2, Table 3, Table 4 and Table 5), the results consistently indicate that performance gains arise from the interaction between a strong initialization and deployment-time adaptation. The CNN–Transformer decoder (Table 2) provides a representation that is better suited to capture both localized SMR-related features and longer-range dependencies than purely local convolutional baselines, which is beneficial under dry-electrode noise and distribution drift. Building on this initialization, session-wise continual adaptation yields progressively improved closed-loop decoding (Table 3) and more coherent class-wise separability in the learned feature space (Table 5, qualitative). Importantly, the physiological analyses (Table 4) show that AO priming elicits stronger SMR modulation in several channel–task pairs, and the sessions/classes exhibiting clearer ERD patterns tend to align with higher decoding reliability. We emphasize that this correspondence provides convergent evidence rather than a causal proof; nevertheless, it strengthens the interpretation that improved online performance reflects better capture of functionally meaningful sensorimotor dynamics rather than solely overfitting to noise.

In our study, the decoder performs four-class motor imagery (MI) classification, where the theoretical chance level is 25%. Therefore, the obtained accuracies, such as 52.78% in offline cross-validation (offline CV) and 66.20% in the online Session 3 experiment, are substantially above chance and demonstrate meaningful decoding capability under a challenging task setting. It is important to note that multi-class MI-BCI systems (e.g., four-command/four-class control), particularly in online closed-loop operation, are considerably more difficult than the commonly studied binary MI paradigms. This is mainly because different imagined movements often produce overlapping EEG features across classes, making reliable classification more challenging. Consequently, stable performance in multi-class MI-BCI systems typically requires substantial user training, system adaptation, and multiple experimental sessions. Furthermore, EEG patterns themselves may evolve during the training process as users gradually learn to modulate their sensorimotor rhythms more effectively. To address these challenges, the present study adopts an action observation (AO) pretraining strategy combined with online fine-tuning, allowing the model to progressively adapt to subject-specific neural patterns and continuously improve decoding performance during online sessions. To further contextualize the contribution of this work, we summarize representative studies on online four-command MI/SMR-based BCI systems. In the work of Hehenberger et al. (2021) [34], a tetraplegic pilot underwent 14 months of mutual training across 26 sessions to perform four-class motor imagery for controlling a racing avatar in the CYBATHLON BCI Race, ultimately achieving an online accuracy of approximately 53%. In another large-scale study, Stieger et al. (2021) [35] released a dataset involving 62 participants, with more than 600 h of EEG recordings, 598 sessions, and over 250,000 trials. In their study, each participant performed four motor imagery tasks (left hand, right hand, both hands, and feet) across multiple sessions to control a 2D cursor task, with an average final control accuracy of approximately 49%. Compared with these studies, the proposed framework incorporates AO-based pretraining data to initialize the model, followed by three online sessions for continual model adaptation. As a result, the online performance shows a progressive improvement from 47.42% in Session 1 to 66.20% in Session 3, demonstrating that the proposed framework effectively narrows the offline-to-online performance gap in a practical real-time four-class MI-BCI scenario.

### 4.4. Limitations and Future Directions

Despite these promising findings, several limitations should be acknowledged. First, the study involved a small cohort of healthy participants and an 8-channel dry-electrode montage, which limits statistical power and generalizability to broader and clinical populations. Second, we observed notable inter-subject variability and class-dependent difficulty (e.g., uneven separability across motor classes), indicating that both user-specific factors and movement-specific characteristics influence closed-loop performance. Third, the online evaluation spanned only three feedback sessions, which is insufficient to establish cross-session generalization across longer time scales or to quantify catastrophic forgetting under extended deployment. Fourth, the current validation focused on a four-class MI paradigm in a controlled VR navigation task, and generalization to richer command sets or less structured environments remains to be demonstrated.

Future work will prioritize (i) larger and more diverse cohorts, including motor-impaired users, (ii) longitudinal and cross-session deployments to quantify drift, robustness, and forgetting, (iii) enhanced personalization (e.g., adaptive kernel sizing or data-driven receptive-field selection) to balance temporal resolution and robustness under dry-EEG, (iv) more robust continual-learning strategies (e.g., regularization- and replay-based updates) to stabilize adaptation under non-stationarity, and (v) optional multimodal extensions (e.g., EMG/kinematics) to further improve reliability.

## 5. Conclusions

This study proposed a dry-electrode, four-class motor-imagery (MI) BCI that combines action–observation-primed training in immersive virtual reality (VR) with domain-incremental continual learning to reduce the offline-to-online performance gap. Across behavioral and neurophysiological evaluations, AO + MI induced stronger sensorimotor ERD than MI alone, providing more informative supervision for initializing a CNN–Transformer decoder. During closed-loop operation, continual adaptation improved online accuracy from 47.4% (Session 1) to 66.2% (Session 3), exceeding the offline cross-validation benchmark (52.8%), while VR navigation time decreased across sessions, indicating enhanced practical controllability through human–machine co-adaptation. Overall, from an expert-systems perspective, the proposed framework serves as an adaptive inference module that updates its internal representation as new evidence becomes available, supporting more reliable decision-making under evolving dry-EEG statistics and advancing toward deployable real-time multi-class BCI control.

## Figures and Tables

**Table 1:** Training Parameters for Pre-trained and Online Continual Learning.

Parameter	Pre-Trained Model	Online Continual-Learning Model
Optimizer	Adam	Adam
Adam betas (*β*1, *β*2)	(0.5, 0.999)	(0.5, 0.999)
Weight decay	1 × 10^−4^	1 × 10^−4^
Epochs	100	20
Batch size	100	16
Base learning rate	3 × 10^−4^	2 × 10^−5^
lr schedule	Warmup + linear decay (Equation (4))	Fixed LR
$n_{warmup}$	10	–

**Table 2:** Summarizes the average performance of five classification models. The proposed CNN–Transformer model achieved the highest accuracy (52.78%) and weighted F1 score (52.21%), outperforming four baseline architectures.

	Proposed (%)		S3T (%)		EEGNet (%)		DeepConvNet (%)		ShallowConvNet (%)	
Subject	Accuracy	Weighted F1	Accuracy	Weighted F1	Accuracy	Weighted F1	Accuracy	Weighted F1	Accuracy	Weighted F1
1	39.56	39.43	39.11	37.45	32.22	31.06	28.78	25.08	33.44	33.40
2	70.22	69.61	64.11	63.43	65.78	65.59	52.44	52.84	68.56	68.62
3	40.56	39.28	38.56	37.36	38.44	37.79	40.22	39.91	39.89	39.59
4	51.44	50.87	46.00	44.96	37.89	38.13	31.89	23.45	47.00	46.88
5	62.11	61.88	47.67	46.28	54.89	54.29	49.56	50.52	59.56	59.20
Average	52.78	52.21	47.09	45.90	45.84	45.37	40.58	38.36	49.69	49.54

**Table 3:** Model performance improved across successive continual-learning sessions.

	Pre-Training (%)		Online Session 1 (%)		Online Session 2 (%)		Online Session 3 (%)	
Subject	Accuracy	Weighted F1	Accuracy	Weighted F1	Accuracy	Weighted F1	Accuracy	Weighted F1
1	39.56	39.43	45.33	45.01	61.67	58.64	54.67	53.12
2	70.22	69.61	69.33	71.06	61.33	60.53	83.33	82.90
3	40.56	39.28	35.79	36.76	52.00	45.34	59.00	59.62
4	51.44	50.87	42.00	39.47	30.53	29.70	65.00	63.15
5	62.11	61.88	44.67	41.83	58.33	54.35	69.00	63.72
Average	52.78	52.21	47.42	46.83	52.77	49.71	66.20	64.50

**Table 4:** (**a**) Comparison of ERD/ERS ratios for left-hand motor imagery across participants S1 to S5 during the pre-training phase. (**b**) Comparison of ERD/ERS ratios for right-hand motor imagery across participants S1 to S5 during the pre-training phase. (**c**) Comparison of ERD/ERS ratios for both-foot motor imagery across participants S1 to S5 during the pre-training phase.

(**a**)						
	**C3 (%)**		**Cz (%)**		**C4 (%)**	
**Subject**	**AO**	**MI**	**AO**	**MI**	**AO**	**MI**
1	15.2	49.0	21.9	44.8	25.7	−1.3
2	−16.6	31.5	−13.1	35.4	−22.0	14.8
3	−29.1	−5.9	−41.1	−22.7	−44.6	−16.9
4	−23.2	−4.9	−19.2	7.6	−24.2	13.4
5	14.9	35.3	0.0	38.6	8.2	13.2
Average	−7.8	21.0	−10.3	20.7	−11.4	4.7
(**b**)						
	**C3 (%)**		**Cz (%)**		**C4 (%)**	
**Subject**	**AO**	**MI**	**AO**	**MI**	**AO**	**MI**
1	36.3	−21.7	46.0	−23.5	28.5	−14.6
2	−27.8	2.0	−21.4	6.7	−23.9	−5.8
3	−7.9	7.7	−19.2	−17.1	−17.5	−17.9
4	−19.5	−22.0	−14.5	−19.7	−13.9	−31.3
5	6.2	23.4	20.1	24.5	2.9	19.5
Average	−2.5	−2.1	2.2	−5.8	−4.8	−10.0
(**c**)						
	**C3 (%)**		**Cz (%)**		**C4 (%)**	
**Subject**	**AO**	**MI**	**AO**	**MI**	**AO**	**MI**
1	7.2	12.2	12.8	20.2	9.1	0.6
2	−24.0	−7.9	−28.5	−10.7	−25.4	−15.5
3	−15.3	−6.6	−34.1	−12.0	−28.1	−8.8
4	−28.6	−16.4	−27.2	−13.4	−28.7	−19.3
5	−10.9	0.5	−10.4	−13.6	−11.9	−10.5
Average	−14.4	−3.6	−17.5	−5.9	−17.0	−10.7

**Table 5:** (**a**) Comparison of ERD/ERS ratios for left-hand motor imagery across participants S1 to S5 during online model adaptation (Online session 1–Online session 3). (**b**) Comparison of ERD/ERS ratios for right-hand motor imagery across participants S1 to S5 during online model adaptation (Online session 1–Online session 3). (**c**) Comparison of ERD/ERS ratios for both-foot motor imagery across participants S1 to S5 during online model adaptation (Online session 1–Online session 3).

(**a**)									
	**C3 (%)**			**Cz (%)**			**C4 (%)**		
**Subject**	**Online Session 1**	**Online Session 2**	**Online Session 3**	**Online Session 1**	**Online Session 2**	**Online Session 3**	**Online Session 1**	**Online Session 2**	**Online Session 3**
1	−3.5	−14.1	−24.2	4.5	−18.8	−20.5	0.1	−11.1	−9.4
2	22.2	−7.6	−33.6	62.4	4.1	−31.5	−3.6	−10.3	−36.0
3	3.1	4.2	−30.5	−0.6	8.3	−20.3	−8.5	−9.8	−14.3
4	−34.6	39.1	−41.8	−42.9	20.9	−41.1	−27.7	30.9	−47.0
5	92.4	−16.5	123.0	115.9	−20.2	164.6	73.5	−12.1	48.6
Average	15.9	1.0	−1.4	27.9	−1.1	10.2	6.8	−2.5	−11.6
(**b**)									
	**C3 (%)**			**Cz (%)**			**C4 (%)**		
**Subject**	**Online Session 1**	**Online Session 2**	**Online Session 3**	**Online Session 1**	**Online Session 2**	**Online Session 3**	**Online Session 1**	**Online Session 2**	**Online Session 3**
1	43.5	23.7	−25.7	47.8	28.6	−20.0	67.1	17.2	−16.4
2	−27.3	−43.4	−16.0	−15.2	−45.6	−10.4	−26.4	−48.9	−19.2
3	−45.1	31.4	−10.2	−49.0	37.5	−10.2	−60.6	24.3	−17.8
4	−19.2	−40.6	−25.2	−26.9	−33.6	−22.5	−15.2	−47.4	−22.7
5	−34.0	23.7	−11.5	−12.6	19.5	16.6	−25.6	20.7	−2.0
Average	−16.4	−1.0	−17.7	−11.2	1.3	−9.3	−12.2	−6.8	−15.6
(**c**)									
	**C3 (%)**			**Cz (%)**			**C4 (%)**		
**Subject**	**Online Session 1**	**Online Session 2**	**Online Session 3**	**Online Session 1**	**Online Session 2**	**Online Session 3**	**Online Session 1**	**Online Session 2**	**Online Session 3**
1	50.6	14.2	3.8	35.1	1.3	0.3	62.4	25.2	13.3
2	77.2	55.9	25.8	71.2	53.9	25.9	66.2	44.7	36.9
3	70.8	14.3	105.0	51.6	−7.8	137.3	65.2	−5.3	132.0
4	−35.7	−9.0	−56.8	−36.5	−15.2	−61.3	−34.0	−20.4	−62.3
5	27.8	−43.0	7.4	50.2	−45.1	16.2	29.4	−44.7	12.7
Average	38.1	6.5	17.0	34.3	−2.6	23.7	37.8	−0.1	26.5

## Data Availability

The original contributions presented in the study are included in the article/Supplementary Materials, further inquiries can be directed to the corresponding author.

## Supplementary Materials

The following supporting information can be downloaded at: https://www.mdpi.com/article/10.3390/bioengineering13050536/s1, Supplementary Table S1: Implementation-oriented qualitative comparison (relative, non-benchmark) of representative architectures. Supplementary Table S2: Architecture hyperparameters of the proposed CNN–Transformer decoder (EEG Conformer) (derived from Figure 8 and the released implementation; numeric Transformer settings for reproducibility). Supplementary Table S3: Pilot-run comparison of representative optimizer and learning-rate schedule configurations used for offline pre-training hyperparameter selection.
