# Supplementary material for: Continual-Learning-Enhanced CNN–Transformer Framework for Real-Time Motor-Imagery BCI in Virtual Environments
Source: Bioengineering (Basel). 2026 May 6;13(5):536. doi: 10.3390/bioengineering13050536 (PMC13203728; doi:10.3390/bioengineering13050536)
Supplement: Supplementary file 1 [file bioengineering-13-00536-s001.zip › bioengineering-4096622-supplementary.pdf]

Supplementary Materials:

Supplementary Table S1: Implementation-oriented qualitative comparison (relative, non-benchmark) of representative architectures.

Aspect	EEGNet (CNN-only)	S3T (CSP-to-Transformer)	Proposed (CNN-Transformer, ours)
Training stability	Generally stable	Can be more sensitive under limited data / drift	Generally stable (CNN front-end + regularization)
Overfitting risk	Typically lower	Potentially higher without strong regularization (attention + small data)	Mitigated via lightweight attention + dropout + online LR schedule
Small-data behavior	Often robust in small-data settings	Can be less robust if CSP mismatch occurs	Best mean Accuracy/F1 among compared baselines under our offline evaluation protocol (Table 2)
Computation	Lower computational footprint (CNN-only)	Higher compute & maintenance overhead (CSP + attention)	Moderate footprint (CNN front-end + lightweight attention)
Interpretability	Relatively interpretable (temporal/spatial filters)	Less straightforward (CSP + attention pipeline)	Moderately interpretable (learned spatial conv + attention weights)
Online update suitability / maintenance	Easy to maintain; may lack global context modeling	CSP may require re-estimation under drift	End-to-end updates compatible with continual adaptation

Supplementary Table S2: Architecture hyperparameters of the proposed CNN–Transformer decoder (EEG Conformer) (derived from Fig. 8 and the released implementation; numeric Transformer settings for reproducibility).

Module	Layer	Hyperparameters
Preprocessing	Input	Raw EEG (8 channels) → epoch input tensor (1×8×500)
Preprocessing	Filtering	1–100 Hz band-pass; 60 Hz band-stop; 4–40 Hz band-pass
Preprocessing	Other	Threshold clipping; epoching; downsampling; z-score normalization
Convolution	Conv2D-1	20 filters; kernel (1×25); stride (1×1); BatchNorm; ELU
Convolution	Conv2D-2	40 filters; kernel (8×1); stride (1×1) (spatial across 8 channels); BatchNorm; ELU
Convolution	AvgPool	kernel (1×75); stride (1×10)
Convolution	Dropout	p = 0.5 (after pooling)
Self-attention	Encoder blocks	N = 3 Transformer encoder blocks (depth) × [MHA + FFN]
Self-attention	Heads	h = 10 attention heads
Self-attention	Embedding	d_model = 40 (equal to CNN output channels)
Self-attention	FFN	2-layer FFN; hidden dim = 160 (forward_expansion = 4 × d_model); dropout p = 0.5
Self-attention	Attention dropout	p = 0.5
Classifier	FC1	Linear(126) + Dropout(p=0.5)
Classifier	FC2	Linear(16) + Dropout(p=0.5)
Classifier	Output	Linear(4) (4-class MI output; Softmax applied for probabilities)

Supplementary Table S3: Pilot-run comparison of representative optimizer and learning-rate schedule configurations used for offline pre-training hyperparameter selection.

ID	Adam (β1, β2)	Weight decay	LR schedule (offline)	lrmax	n_warmup	Batch size	Validation trend*	Convergence stability†	Used in main experiments
**A (selected)**	**(0.5, 0.999)**	**1×10^{-4}**	**Warmup + linear decay (Eq. (4))**	**3×10^{-4}**	**10**	**100**	**Best**	**Stable**	**Yes**
B	(0.5, 0.999)	1×10^{-4}	No warmup, constant lr	3×10^{-4}	0	100	Lower	Early oscillation in some folds	No
C	(0.9, 0.999)	1×10^{-4}	Warmup + linear decay (Eq. (4))	3×10^{-4}	10	100	Slightly lower	Stable	No
D	(0.5, 0.999)	1×10^{-4}	Warmup + linear decay (Eq. (4))	1×10^{-3}	10	100	Lower	Unstable / occasional divergence	No
